# Socioeconomic inequalities in health behaviours pre- and post-COVID-19 among Japanese school-aged adolescents: a nationally representative three-wave repeated cross-sectional survey

**DOI:** 10.1265/ehpm.25-00052

**Published:** 2025-09-11

**Authors:** Akira Kyan, Minoru Takakura

**Affiliations:** 1 Faculty of Medicine, University of the Ryukyus, 1076 Kiyuna, Ginowan City, Okinawa 901-2720, Japan; 2 Center for Social Common Capital beyond 2050, Institutional Advancement and Communications, Kyoto University; 3 Graduate School of Sports and Health Sciences, Meio University, Nago City, Okinawa, Japan

**Keywords:** Socioeconomic inequalities, COVID-19 pandemic, Adolescent health behaviours, Physical activity, Screen time, Breakfast consumption

## Abstract

**Background:**

Changes in socioeconomic inequalities in health behaviours following the COVID-19 pandemic remain unknown, particularly among Japanese school-aged adolescents. Therefore, in this study, we examined changes in socioeconomic inequalities in school-aged adolescents’ health behaviours, including physical activity (PA), screen time (ST), sleep duration, breakfast consumption, and bowel movement frequency, before and after the pandemic.

**Methods:**

This three-wave repeated cross-sectional study utilised data from the 2019, 2021, and 2023 National Sports-Life Survey of Children and Young People in Japan, analysing data from 766, 725, and 604 participants aged 12–18 years, respectively. Favourable health behaviours were defined as moderate-to-vigorous PA of ≥60 min/day, ST <2 h/day, sleep duration of 8–10 h, daily breakfast consumption, and bowel movements at least every 3 days. Absolute and relative socioeconomic inequalities were quantified using the slope index of inequality (SII) and the relative index of inequality (RII). Temporal changes were analysed using quadratic trend analyses, employing interaction terms between socioeconomic status and survey year.

**Results:**

Significant quadratic trends indicated that socioeconomic inequalities in breakfast consumption decreased substantially from 2019 (SII: 20.7%, RII: 5.09) to 2021 (SII: −0.1%, RII: 0.95) but resurged in 2023 (SII: 16.2%, RII: 3.70). This resurgence may have been primarily driven by changes among those in the moderately low-income (poverty level II) and higher-income groups, which had a breakfast consumption rate of 81.0, 87.0, and 76.4% in 2019, 2021, and 2023, and 88.7, 82.1, and 87.5%, respectively. Among low-income households, adherence to PA recommendations significantly declined from 18.6% to 5.3%, and ST adherence worsened over the study period. No significant inequalities or trends were observed for sleep duration or bowel movement frequency across survey years.

**Conclusions:**

Socioeconomic disparities in breakfast consumption among Japanese school-aged adolescents resurfaced after initially narrowing during the pandemic, likely driven by changes in moderately low-income and higher-income groups. Among low-income households, adherence to PA and ST guidelines declined over time. However, at the population level, socioeconomic inequalities in PA did not exhibit a consistent trend of widening or narrowing. This study highlights the need for sustained public health initiatives to address these socioeconomic disparities.

**Supplementary information:**

The online version contains supplementary material available at https://doi.org/10.1265/ehpm.25-00052.

## Graphical Abstract

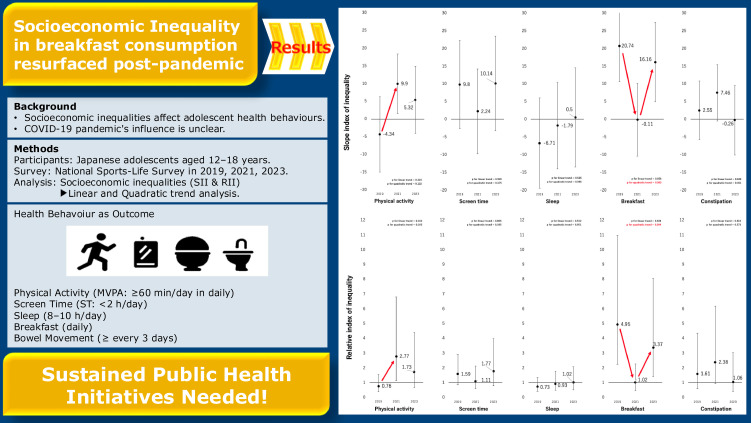

## Background

The World Health Organization (WHO) reported over 770 million confirmed COVID-19 cases and seven million deaths globally by December 2024 [1], resulting in an unprecedented global disruption. The pandemic has significantly impacted lifestyles and health behaviours, particularly among youth. Despite improvements in certain health behaviours owing to societal changes attributable to the pandemic, such as better diet quality [2, 3], other behaviours, including physical activity (PA) [4], screen time (ST) [5], and sleep patterns and quality [6, 7], have reportedly deteriorated.

The limited evidence on the differential impact of the pandemic on vulnerable subgroups, particularly those defined by socioeconomic status, is particularly concerning [4, 6–8]. Well-documented inequalities in fundamental health behaviours among adolescents, such as PA [9], ST [10], sleep patterns [11], bowel movement frequency [12], and food intake [13], vary with family or neighbourhood economic status even prior to the COVID-19 pandemic onset. However, these socioeconomic inequalities have widened following the pandemic. For example, Scottish adolescents [14] exhibited decreased levels of PA, while American adolescents from low-income households experienced poor sleep patterns [6]. Even in Japan, where COVID-19 policy restrictions were relatively lenient [15], infection control measures, such as restrictions on restaurant operations, cancellation of large-scale events, and limitations on inter-prefectural travel [16], led to significant economic inequalities across occupations. For example, the service industry experienced a substantial decline in revenue, resulting in widespread unemployment and bankruptcies. In contrast, the information and communication sector, including online businesses, witnessed increased revenues [16]. Data from representative Japanese adults indicate that the socioeconomic inequalities in PA have become more pronounced during the COVID-19 pandemic [17]. These economic disparities, combined with behavioural restrictions such as social distancing, may have affected health inequalities. For example, a previous study using national survey data indicated widening inequalities in PA and narrowing inequalities in breakfast consumption among Japanese school-aged adolescents [18].

In response to global trends, declining number of severe cases, and decreased incidence of new infections, Japan eased the COVID-19-related legal restrictions on 8 May 2023. Since before the COVID-19 pandemic, Japanese schools have been promoting healthy behaviour through the slogan ‘Hayane Hayaoki Asagohan’ (‘early bedtimes, early risings, and breakfast’) [19]. Similarly, countries such as Canada [20] and Australia [21] have published guidelines as part of global awareness campaigns advocating adherence to recommended levels of a combination of PA, ST, and sleep. Furthermore, the frequency of bowel movements serves as a useful indicator of dietary habits, intestinal environment, digestive organ health, and overall health. It is non-invasive, inexpensive, and can be monitored daily, making it an important health behaviour to monitor in epidemiological studies [22–24].

Understanding the evolution of socioeconomic inequalities in these health behaviours is crucial for evaluating the effectiveness of current policies and shaping future directions. Therefore, in this study, we aimed to elucidate trends in socioeconomic inequalities in health behaviours before and after the COVID-19 pandemic among Japanese school-aged adolescents.

## Methods

### Data source

We used data from the 2019, 2021, and 2023 National Sports-Life Survey of Children and Young People conducted by the Sasakawa Sports Foundation [25]. Each year, a sample was selected using two-stage stratified random sampling from 225 locations, proportionally distributed by district and city size based on population data from the Basic Resident Register of the preceding year: 2019: 61 metro areas, 94 and 50 cities with a population of more than 100,000 and less than 100,000, respectively, and 20 towns/villages; 2021: 61 metro areas, 96 and 50 cities with a population of more than 100,000 and less than 100,000, respectively, and 18 towns/villages; 2023: 63 metro areas, 95 and 49 cities with a population of more than 100,000 and less than 100,000, respectively, and 18 towns/villages. The primary sampling unit was based on the 2015 census study area. The sample size per location ranged from 10 to 19 participants. Survey points were selected using probability-proportional-to-size sampling. For strata allocated two or more survey points, the sampling interval was calculated as follows:

$$\begin{array}{rl} & \text{Sampling interval}\\ & =\frac{\text{Total number of people aged 4–21 years in the stratum}}{\text{Number of survey sites allocated to the stratum}} \end{array}$$

The total sample included 3,000 individuals. Participants were informed that the data would be used for academic research and provided informed consent. The survey was conducted using the visit-leave-collect method. Researchers visited the homes of respondents to distribute questionnaires, requested their completion within the survey period, and then revisited to collect the completed questionnaires. Data were collected through self-administered questionnaires completed by adolescents and their parents/guardians. If a respondent was unable to understand or complete the questionnaire independently, the interviewer read the questions and recorded their responses. Survey periods were between June and July of each survey year. Detailed descriptions of the survey methodology are available on the Sasakawa Sports Foundation website [18, 25].

This study focused on respondents aged 12–21 years, with 1,675 (55.8%) in 2019, 1,663 (55.4%) in 2021, and 1,495 (49.8%) in 2023. For analysis, only participants aged 12–18 years were included, excluding 18-year-olds not enrolled in high school. In Japan, most high school students are aged 15–18, and 17- and 18-year-olds in the third grade share similar school routines. If attending high school, individuals aged 16, 17, and 18 years generally share similar lifestyles and can therefore be considered a group with comparable attributes.

Thus, 18-year-olds attending high school were included in the analysis; however, the age range for assessing PA, ST, and sleep recommendations was limited to 17 years. The final sample included 1,076 (506 girls) in 2019, 1,025 (508 girls) in 2021, and 898 (448 girls) in 2023. After excluding participants with missing data, the analysis sample included 766 (407 girls) in 2019, 725 (360 girls) in 2021, and 604 (305 girls) in 2023.

The Institutional Review Board of the University of the Ryukyus determined that the research was eligible for exemption, as the identities of participants could not be determined from the data.

### Measures

#### Physical activity

PA was assessed using the Japanese version of the PA questionnaire in the Health Behaviour in School-aged Children survey [26], which is widely used for monitoring moderate-to-vigorous PA in adolescents [27]. The sample questionnaire is available in the previous study that reported its validity [28]. PA was defined as any activity that increased heart rate and induced breathlessness for a period, including walking to school, riding a bicycle, playing outside, walking fast, running, sports such as swimming, dancing, roller skating, skateboarding, surfing, soccer, and basketball. Participants were asked: ‘Over the past 7 days, on how many days were you physically active for a total of at least 60 min per day?’ Respondents selected their answers from options ranging from 0 to 7 days. Based on the WHO guidelines on PA and sedentary behaviours [29], participants were dichotomized as “active” if they reported being physically active for at least 60 minutes on all 7 days, and “inactive” otherwise.

#### Screen time

ST was measured by assessing recreational TV/DVD viewing time and computer/game/smartphone usage separately for weekdays and weekends. The participants were asked: ‘For how many hours per day do you watch TV or DVDs or use computers, video games (including TV, computer, and cellular device games), or smartphones outside of school and/or work?’ Response options included: ‘less than 30 min/day’, ‘30 min to 1 h/day’, ‘1–2 h/day’, ‘2–3 h/day’, ‘3–4 h/day’, ‘4–5 h/day’, ‘more than 5 h/day’, and ‘I do not know’. Responses were converted to minutes using the midpoint method, with ‘I do not know’ categorised as missing data. The average daily ST was calculated using the following formula:

$$\begin{array}{rl} & ([\text{minutes of ST on weekdays}\times 5]\\ & \;+[\text{minutes of ST on weekends}\times 2])/7. \end{array}$$

The participants were classified using a cut-off of 2 h of recreational ST, based on the WHO guidelines [29]. This method is aligned with those reported in previous studies, which used time spent on TV, smartphones, tablets, and PCs as ST indicators [30].

#### Sleep duration

Sleep duration was calculated based on self-reported bedtimes and wake-up times for weekdays and weekends. The participants provided typical sleep and wake times for each. The average daily sleep duration was calculated as follows:

$$\begin{array}{rl} & ([\text{sleep duration on weekdays}\times 5]\\ & \;+[\text{sleep duration on weekends}\times 2])/7. \end{array}$$

Participants were classified based on whether their sleep duration fell within the recommended range of 8–10 h per night by the National Sleep Foundation [31].

#### Breakfast consumption

Breakfast frequency was determined by asking: ‘How often do you have breakfast per week?’ Responses included: ‘almost every day’, ‘4–5 days’, ‘2–3 days’, and ‘very few’. Participants were dichotomised into ‘almost every day’ or ‘others’, following the recommendations of Japan’s Ministry of Agriculture, Forestry, and Fisheries [32].

#### Bowel movement frequency

Bowel movement frequency was assessed using the question: ‘How often do you have a bowel movement?’ Response options included: ‘almost every day’, ‘once every 2 days’, ‘once every 3 days’, ‘less than once every 3 days’, and ‘irregularly’. The participants were categorised into ‘almost every day to once every 3 days’ or ‘less than once every 3 days and irregular’, based on the definition of constipation [12].

#### Household income

Household income was reported by parents/guardians of the participants using 11 options, ranging from ‘no income’ to ‘10 million yen or more’. Responses of ‘I do not know’ were treated as missing data. The midpoint of each range was used to calculate equivalent household income, which was adjusted by dividing the total household income by the square root of the number of household members [22]. The income levels were categorised into three groups (I, II, and non-poverty households) based on one-half of the median equivalent household income in each survey year, with level I representing the most economically disadvantaged group [33]. The classification of poverty levels by survey year (values expressed in units of one million yen) was as follows: Poverty I was defined as values less than 1.453 million yen in 2019, less than 1.531 million yen in 2021, and less than 1.588 million yen in 2023. Poverty II encompassed values ranging from 1.453 to 2.907 million yen in 2019, 1.531 to 3.062 million yen in 2021, and 1.588 to 3.175 million yen in 2023. Non-poverty households included values greater than 2.907 million yen in 2019, exceeding 3.062 million yen in 2021, and greater than 3.175 million yen in 2023. One million yen corresponded to approximately 14,000 US dollars at the survey time.

### Covariates

Covariates included place of residence, family structure, sex, age, sports participation, self-rated health, and preference for PA, which were all considered potential confounders [9, 34]. The place of residence was categorised by the population size, with a threshold of 100,000 residents. Family structure was coded as ‘lived with both parents’ or ‘other’. Sports participation was assessed by the participants if they engaged in extracurricular exercise activities in school, local sports clubs, or private settings. Self-rated health was dichotomised as ‘good’ or ‘poor’, and preference for PA was categorised as ‘liked’ or ‘disliked’.

### Statistical analyses

The prevalence of health behaviours was calculated for each income level and survey year. The changes over time in each health behaviour by income level in 2019 and 2021, and 2021 and 2023, were confirmed using the chi-square test. Temporal trends across income levels were analysed using the Cochran–Armitage test for trend. Socioeconomic inequalities between low- and high-income groups were evaluated using absolute and relative measures, with 95% confidence intervals (CIs) for each income level.

For absolute measures, the slope index of inequality (SII) [35–37] was calculated using generalised linear models with a binomial distribution and an identity link function. When the binomial model did not converge, a model with a normal distribution and an identity link was applied [36]. For relative measures, the relative index of inequality (RII) [35–37] was calculated using generalised linear models with a binomial distribution and a log link function. Both indices used ridit scores for income levels as independent variables, quantifying inequality between the theoretical lowest and highest income groups while accounting for the cumulative income distribution [37]. SII quantifies the absolute difference in prevalence across income levels and is useful for understanding the potential public health burden. In contrast, RII reflects proportional inequality, providing valuable insights from an equity and policy perspective. Together, these indices offer a comprehensive interpretation of socioeconomic disparities. SII reflects the absolute gap in health behaviours between the most and least advantaged groups, directly relating to the number of individuals affected and therefore carrying important implications for population-level burden and public health planning. In contrast, RII expresses the proportional difference, highlighting the degree of inequality of the distribution, regardless of the overall prevalence of the behaviour. Therefore, RII is particularly more informative for assessing equity and fairness. For example, SII can indicate large RII) for rare behaviours, whereas prevalent behaviours may yield small RIIs despite large absolute differences. By reporting both indices, we aimed to provide a more nuanced understanding of the trends, recognising that the public health impact and the social equity implications of inequalities may not always align.

To assess whether inequalities had recovered by 2023, a quadratic time-trend model was constructed. A linear trend was first examined for consistent increases or decreases, followed by a quadratic trend to capture directional changes such as levelling off or reversing. The model was specified as follows:

$$\begin{array}{rl} Y & =\beta_{0}X_{\text{intercept}}+\beta_{1}X_{\text{ridit\_score}}+\beta_{2}X_{\text{survey year}}\\ & \;+\beta_{3}X_{{\text{survey year}}^{2}}+\beta_{4}X_{\left(\text{ridit\_score}\times {\text{survey year}}^{2}\right)}\\ & \;+\beta_{\text{n}}X_{\text{covariates}}\ldots +\epsilon , \end{array}$$

where Y represents the outcome variable and ϵ denotes the error term. The survey year was coded as 1 for 2019, 2 for 2021, and 3 for 2023 [33]. The Wald test was used to assess the significance of interaction terms, with covariates included in all models to control for confounders.

Trends were interpreted based on the guidelines of the Centres for Disease Control and Prevention [38]. A significant linear trend indicated consistent increases or decreases. A significant quadratic trend alone suggested no overall linear change but indicated directional shifts. Significant linear and quadratic trends together suggested an overall linear pattern with specific changes, such as levelling off or reversing.

## Results

Table 1 presents the distribution of the participants by sociodemographic characteristics across survey years. Survey year and compliance with PA and ST guidelines were significantly associated (*p* < 0.001). PA prevalence was significantly lower in 2021 than in other years, whereas ST decreased in 2021 and 2023. Missing data proportions remained consistent across surveys (Additional file 1). Household income measure had the highest proportion of missing data (22.2–25.8%), as ‘I do not know’ responses were treated as missing. Among the participants who met the age inclusion criteria, demographic factors, such as age, sex, or residence area, did not differ significantly between individuals included in the analysis and those excluded from it. For the variables of interest, differences in income and PA were only noted in 2019. Individuals with lower income and non-compliance with PA recommendations were more likely to be excluded.

**Table 1:** Distribution of study participants by sociodemographic characteristics

	**2019**		**2021**		**2023**		***p* ^a^**
		
	**n**	**%**	**n**	**%**	**n**	**%**	
Total	765	36.5	725	34.6	604	28.8	
Sex							
Male	358	46.8	365	50.3	299	49.5	0.361
Female	407	53.2	360	49.7	305	50.5	
Age group (year)							
12	88	11.5	78	10.8	84	13.9	0.126
13	152	19.9	129	17.8	88	14.6	
14	122	15.9	110	15.2	110	18.2	
15	140	18.3	120	16.6	107	17.7	
16	119	15.6	133	18.3	85	14.1	
17	114	14.9	120	16.6	96	15.9	
18	30	3.9	35	4.8	34	5.6	
Residence area							
metropolises & cities with population 100,000+	678	88.6	652	89.9	545	90.2	0.575
Cities with population <100,000 & Towns & villages	87	11.4	73	10.1	59	9.8	
Equivalent household income (million yen)							
Poverty I	86	11.2	90	12.4	75	12.4	0.929
Poverty II	290	37.9	277	38.2	233	38.6	
Non-poverty households	389	50.8	358	49.4	296	49.0	
Family structure							
Both parents	650	85.0	635	87.6	532	88.1	0.175
other	115	15.0	90	12.4	72	11.9	
Sports participation							
No	496	64.8	433	59.7	373	61.8	0.122
Yes	269	35.2	292	40.3	231	38.2	
Self-rated health							
Good	650	85.0	602	83.0	491	81.3	0.192
Bad	115	15.0	123	17.0	113	18.7	
Preference of physical activity							
Dislike	142	18.6	130	17.9	124	20.5	0.461
Like	623	81.4	595	82.1	480	79.5	
MVPA							
<7 days	611	79.9	640	88.3	524	86.8	<0.001
7 days	154	20.1	85	11.7	80	13.2	
Screen time							
≥2 h	589	77.0	617	85.1	520	86.1	<0.001
<2 h	176	23.0	108	14.9	84	13.9	
Adequate sleep duration							
8–10 h	279	36.5	253	34.9	232	38.4	0.416
other	486	63.5	472	65.1	372	61.6	
Breakfast							
Almost everyday	640	83.7	604	83.3	495	82.0	0.686
Other	125	16.3	121	16.7	109	18.0	
Constipation							
Yes	77	10.1	76	10.5	72	11.9	0.524
No	688	89.9	649	89.5	532	88.1	

a: χ^2^ test

*Poverty I* 2019: <1.453, 2021: <1.531, 2023: <1.588

*Poverty II* 2019: 1.453–2.907, 2021: 1.531–3.062, 2023: 1.588–3.175

*Other* 2019: >2.907, 2021: >3.062, 2023: >3.175

Table 2 summarises the prevalence of each health behaviour by income level and survey years. Significant changes in adherence to health behaviour guidelines between 2019 and 2021 or between 2021 and 2023 were observed for PA, ST, and breakfast consumption. Adherence to the PA guidelines significantly declined from 2019 to 2021 across all income levels (Poverty I: *p* = 0.003, Poverty II: *p* = 0.002, Non-poverty households: *p* = 0.029), with no significant changes observed from 2021 to 2023. Declining trends in PA across the three waves were observed among participants in poverty levels I and II. Although income-related inequalities in PA widened in 2021, they were no longer significant by 2023. For both ST and daily breakfast consumption, adherence significantly declined from 2019 to 2021 in the non-poverty group (screen time: p < 0.001; breakfast: *p* = 0.011), whereas a significant decline from 2021 to 2023 was observed in the poverty level II group (screen time: *p* = 0.013; breakfast: *p* = 0.002). For ST, declining trends were observed in poverty level II and non-poverty households across the waves. By 2023, the gap between poverty level II and non-poverty households was highly significant. Breakfast consumption exhibited no consistent temporal trend by income level. However, in 2023, the prevalence was significantly lower in poverty level II than in other poverty levels. The proportion of poverty level II was lower than that of poverty level I in 2023. These differences by socioeconomic levels were not observed for sleep duration and bowel frequency.

**Table 2:** Prevalence of health behaviour in 2019, 2021, and 2023 according to the equivalent household income

	**Physical activity^a^**									**Screen time^b^**								
	
	**2019**		**2021**		**2023**		***p*^f^ for 2019–2021**	***p*^f^ for 2021–2023**	***p*^g^ for ** **trend**	**2019**		**2021**		**2023**		***p*^f^ for 2019–2021**	***p*^f^ for 2021–2023**	***p*^g^ for ** **trend**
					
	**n**	**(%)**	**n**	**(%)**	**n**	**(%)**				**n**	**(%)**	**n**	**(%)**	**n**	**(%)**			
Total	154	20.1	85	11.7	80	13.2				176	23.0	108	14.9	84	13.9			
Equivalent household income (million yen)																		
Poverty I	16	18.6	4	4.4	4	5.3	0.003	0.791	0.001	16	18.6	12	13.3	10	13.3	0.339	1.000	0.256
Poverty II	59	20.3	30	10.8	32	13.7	0.002	0.318	0.029	66	22.8	47	17.0	22	9.4	0.084	0.013	0.004
Non-poverty households	79	20.3	51	14.2	44	14.9	0.029	0.823	0.063	94	24.2	49	13.7	52	17.6	<0.001	0.172	0.002
P for Fisher’s exact test		0.932		0.030		0.090					0.537		0.467		0.027			

	**Sleep**									**Breakfast^d^**								
	
	**2019**		**2021**		**2023**		***p*^f^ for 2019–2021**	***p*^f^ for 2021–2023**	***p*^g^ for trend**	**2019**		**2021**		**2023**		***p*^f^ for 2019–2021**	***p*^f^ for 2021–2023**	***p*^g^ for trend**
					
	**n**	**(%)**	**n**	**(%)**	**n**	**(%)**				**n**	**(%)**	**n**	**(%)**	**n**	**(%)**			

Total	279	36.5	253	34.9	232	38.4				640	83.7	604	83.3	495	82.0			
Equivalent household income (million yen)																		
Poverty I	33	38.4	29	32.2	30	40.0	0.393	0.299	0.888	60	69.8	69	76.7	58	77.3	0.301	0.919	0.289
Poverty II	108	37.2	105	37.9	88	37.8	0.870	0.974	0.688	235	81.0	241	87.0	178	76.4	0.053	0.002	0.263
Non-poverty households	138	35.5	119	33.2	114	38.5	0.521	0.161	0.634	345	88.7	294	82.1	259	87.5	0.011	0.058	0.737
P for Fisher’s exact test		0.829		0.403		0.941					<0.001		0.051		0.002			

	**Constipation^e^**																	

	**2019**		**2021**		**2023**		***p*^f^ for 2019–2021**	***p*^f^ for 2021–2023**	***p*^g^ for ** **trend**									
		
	**n**	**(%)**	**n**	**(%)**	**n**	**(%)**												

Total	77	10.1	76	10.5	72	11.9												
Equivalent household income (million yen)																		
Poverty I	5	5.8	6	6.7	9	12.0	0.815	0.235	0.285									
Poverty II	36	12.4	30	10.8	28	12.0	0.557	0.674	0.900									
Non-poverty households	36	9.3	40	11.2	35	11.8	0.386	0.795	0.320									
P for Fisher’s exact test		0.152		0.446		0.997												
	

Total number of 2019, 2021, and 2023: 765, 725, and 604

Poverty I: 2019: <1.453; 2021: <1.531; 2023: <1.588

Poverty II: 2019: 1.453–2.907; 2021: 1.531–3.062; 2023: 1.588–3.175

Non-poverty households: 2019: 2.907+; 2021: 3.062+; 2023: 3.175+

a engaging in ≥60 min of moderate-to-vigorous physical activity per day on all 7 days.

b engaging in less than 2 h of recreational screen time per day.

c having 8–10 h of sleep per night.

d eating breakfast almost every day.

e defecation frequency is less than once every 3 days.

f Chi-square test

g Cochran-Armitage trend test

Table 3 presents the SII and RII for each health behaviour across survey years. Significant quadratic trends were observed only for breakfast consumption in both the SII and RII. The quadratic trends were positive (SII: coefficient = 0.216, *p* = 0.002; RII: coefficient = 1.519, *p* = 0.002). Between 2019 and 2021, the SII for breakfast consumption decreased from 21.63% (95% CI: 11.3–32.0) to −0.63% (95% CI: −11.5–10.3) and became nonsignificant. However, the SII regained significance in 2023, with an observed increase to 16.16% (95% CI: 8.6–31.9). Similarly, the RII became insignificant between 2019 and 2021, decreasing from 5.09 (95% CI: 2.39–10.84) to −0.63 (95% CI: −10.4–10.2), but regained significance in 2023 at 3.7 (95% CI: 1.7–8.0). This trend remained consistent after adjusting for covariates.

**Table 3:** Health behaviour inequalities (SII & RII) by income levels and survey year

	**2019**		**2021**		**2023**		**Linear trend^c^**		**Quadratic trend^c^**	
				
	**index**	**95% CI**	**index**	**95% CI**	**index**	**95% CI**	**Coefficient**	***p***	**Coefficient**	***p***
Physical activity										
Crude SII	1.23	−9.61, 12.07	12.31	4.39, 20.22	9.79	0.53, 19.05	0.036	0.311	−0.068	0.211
Adjusted SII^a,b^	−4.34	−14.96, 6.28	9.90	1.47, 18.33	5.32	−4.08, 14.73	0.044	0.224	−0.083	0.132
Crude RII	1.08	0.55, 2.12	3.15	1.31, 7.57	2.18	0.91, 5.25	0.460	0.141	−0.718	0.175
Adjusted RII^a^	0.76	0.37, 1.55	2.77	1.13, 6.81	1.73	0.68, 4.41	0.524	0.102	−0.873	0.105
Screen time										
Crude SII	15.33	2.64, 28.03	0.51	−11.59, 12.62	15.07	1.96, 28.18	0.000	0.993	0.147	0.058
Adjusted SII^a,b^	9.80	−2.69, 22.3	2.24	−9.77, 14.26	10.14	−3.19, 23.47	−0.005	0.920	0.103	0.175
Crude RII	1.96	1.10, 3.49	1.03	0.55, 1.90	2.38	1.14, 5.00	0.043	0.856	0.746	0.060
Adjusted RII^a^	1.59	0.86, 2.93	1.11	0.58, 2.13	1.77	0.79, 3.98	0.042	0.865	0.540	0.195
Sleep duration										
Crude SII	−4.09	−17.22, 9.04	−4.07	−17.24, 9.09	−0.41	−15.26, 14.44	0.017	0.729	0.018	0.828
Adjusted SII^a,b^	−6.71	−19.49, 6.07	−1.79	−13.99, 10.41	0.50	−13.49, 14.49	0.029	0.545	0.005	0.946
Crude RII	0.84	0.48, 1.48	0.84	0.47, 1.49	0.98	0.52, 1.84	0.078	0.721	0.079	0.829
Adjusted RII^a^	0.73	0.40, 1.34	0.93	0.48, 1.77	1.02	0.50, 2.09	0.145	0.532	0.024	0.951
Breakfast										
Crude SII	21.63	11.30, 31.96	−0.63	−11.54, 10.27	20.28	8.62, 31.93	−0.021	0.598	0.216	0.002
Adjusted SII^a,b^	20.74	10.61, 30.88	−0.11	−10.38, 10.15	16.16	4.94, 27.38	−0.017	0.656	0.197	0.003
Crude RII	5.09	2.39, 10.84	0.95	0.43, 2.09	3.70	1.71, 8.02	−0.197	0.470	1.519	0.002
Adjusted RII^a^	4.95	2.23, 10.96	1.02	0.46, 2.27	3.37	1.41, 8.06	−0.146	0.608	1.471	0.004
Constipation										
Crude SII	−0.97	−8.70, 6.77	3.93	−4.23, 12.09	−0.34	−10.21, 9.53	0.006	0.855	−0.057	0.273
Adjusted SII^a,b^	2.55	−5.75, 10.85	7.46	−0.45, 15.38	−0.26	−10.04, 9.52	−0.007	0.828	−0.039	0.451
Crude RII	0.90	0.38, 2.10	1.53	0.63, 3.74	0.97	0.38, 2.47	0.044	0.893	−0.494	0.376
Adjusted RII^a^	1.61	0.59, 4.36	2.38	0.92, 6.19	1.06	0.37, 3.05	−0.085	0.810	−0.331	0.573

CI, confidence interval, RII relative index of inequality, SII slope index of inequality (%)

Poverty I: 2019: <1.453; 2021: <1.531; 2023: <1.588

Poverty II: 2019: 1.453–2.907; 2021: 1.531–3.062; 2023: 1.588–3.175

Non-poverty households: 2019: 2.907+; 2021: 3.062+; 2023: 3.175+

a Adjusted for place of residence, family structure, sex, age, sports participation, self-rated health, and preference of PA. To explore the association between the outcomes, when each health behaviour was considered in the dependent variable as an outcome, other health behaviours were included as independent variables.

b Generalised linear model with normal distribution and identity link function

c Wald test of the interaction term between equivalent household income (ridit score) and survey year. Linear and quadratic trend coefficients for SII indicate the estimated annual (linear) and annual squared (quadratic) changes in percentage points of the SII across the socioeconomic gradient (ridit scale from 0 to 1).

For PA, the linear and quadratic trends were not significant. However, between 2019 and 2021, the SII increased from 1.23% (95% CI −9.61–12.07) to 12.31% (95% CI 4.4–20.2) and RII increased from 1.08 (95% CI 0.6–2.1) to 3.15 (95% CI 1.3–7.6). Although both the SII and RII for 2023 were relatively high (SII: 9.79%, 95% CI: 0.5–19.1; RII: 2.18, 95% CI: 0.5–4.8), they did not reach statistical significance. Both the SII and RII for ST were significant in 2019 at 15.33% (95% CI: 2.64–28.03) and 1.96 (95% CI: 1.1–3.5), respectively, in the crude model. However, these indices lost significance in 2021, declining to 0.51% (SII, 95% CI: −11.6–12.6) and 1.03 (RII, 95% CI: 0.6–1.9). In 2023, both indices regained statistical significance (SII: 15.07%, 95% CI: 1.7–28.4; RII: 1.96, 95% CI: 1.1–3.5). Nevertheless, in the adjusted model, neither the SII nor the RII for ST reached statistical significance. No significant income-related inequalities or temporal trends were observed for sleep duration or bowel movement frequency across the survey years. The results of the linear and quadratic trend model fitting are presented in e-table 2. The difference between the two models was minimal for all health behaviours.

## Discussion

To the best of our knowledge, this is the first study to investigate time trends in socioeconomic inequalities in fundamental health behaviours among Japanese school-aged adolescents before and after the COVID-19 pandemic. Our findings revealed a significant quadratic trend in breakfast consumption, indicating a resurgence in socioeconomic inequality. The SII and RII values (Table 3), which incorporate population distribution across income groups, serve as robust measures of absolute and relative socioeconomic inequalities. Although these indices became statistically nonsignificant in 2021, indicating a temporary narrowing of inequalities, they regained statistical significance by 2023. This resurgence in breakfast skipping was primarily driven by decreased breakfast consumption among moderately low-income households (poverty level II) and increased prevalence among higher income households (Table 2). A systematic review of dietary pattern changes among youth during the COVID-19 pandemic revealed mixed results for breakfast habits with six studies noting improvement and five studies indicating deterioration [2]. Our findings suggest that this variability is partly attributable to heterogeneity by socioeconomic status.

Our results offer practical implications. Given that the overall prevalence of daily breakfast consumption remained relatively high, even among low-income school-aged adolescents, further reductions in absolute inequality (SII) may be inherently limited. In contrast, the persistence of relative inequality (RII) suggests that targeted interventions for disadvantaged subgroups remain necessary. To promote both equity and effectiveness, a dual strategy may be warranted: (1) sustaining population-wide promotion efforts to maintain high baseline adherence, and (2) intensifying support for vulnerable groups to close the remaining proportional gaps. This approach aligns with equity-oriented public health implementation frameworks and offers a pathway to more inclusive and sustained behavioural improvements.

In Japan, low-income households are often associated with employment in non-regular jobs characterised by long working hours and low wages, such as positions in convenience stores, food service, and transportation [39–41]. During the pandemic, many of these sectors faced economic disruptions [16], resulting in reduced working hours or temporary unemployment for many parents employed in these occupations. Consequently, these families experienced increased time at home, indirectly providing school-aged adolescents more opportunities to have breakfast; however, this remains speculative and further research is required to confirm it. Moreover, infection prevention measures, such as temporary class closures and staggered school attendance [42], provided school-aged adolescents with enhanced flexibility in their morning routines, further facilitating regular breakfast consumption [43]. As economic activities gradually resumed post-pandemic, parental employment conditions and school routines also reverted, likely leading the breakfast patterns of school-aged adolescents to return toward pre-pandemic levels. To our knowledge, no studies have yet examined dietary patterns following the conclusion of the pandemic; thus, future research is needed to explore longer-term changes in dietary behaviours beyond breakfast consumption [3].

Socioeconomic inequalities in PA widened between 2019 and 2021 and persisted through 2023. The lack of a significant quadratic trend suggests that, after the initial widening, these inequalities have remained stable rather than returning to pre-pandemic levels. This widening of inequalities can be attributed to the suspension of extracurricular activities, involving two-thirds of school-aged adolescents [44], during the pandemic, and the cancellation of sporting events to prevent infection spread [18]. The relaxation of such restrictions may have narrowed or eliminated these gaps.

The overall compliance with PA and ST guidelines worsened. A nationwide survey by Japan’s Ministry of Education revealed reduced exercise and increased ST among middle school students [44]. A repeated cross-sectional study in Okinawa also revealed decreased adherence to WHO PA guidelines and increased ST during the pandemic [45]. These findings in Japan also align with previous findings from a meta-analysis including data from Europe, North America, South America, and Asia [4, 5]. In the present study, although compliance with PA guidelines improved for low-income groups, ST significantly worsened. These data highlight the importance of continuously monitoring socioeconomic inequalities in ST.

This study has some limitations. Firstly, the cross-sectional design of the study prevents tracking individual trajectories over time. Secondly, although the sampling method ensured data representativeness, eligible sample proportions were 45.7, 43.6, and 40.4% in 2019, 2021, and 2023, respectively. However, no significant differences in demographic factors were observed across survey years. Additionally, the distribution of participants across districts and cities closely mirrored the national population structure. Thirdly, the proportion of missing income data was relatively high but remained consistent across survey years, mitigating concerns about systematic bias. The increased proportion of low-income and low PA levels among excluded participants might have led to an underestimation of the income-PA association in 2019.

## Conclusions

This study highlights a resurgence of socioeconomic inequalities in breakfast consumption among Japanese school-aged adolescents, which had temporarily narrowed during the COVID-19 pandemic. This resurgence may have been partly attributable to decreased adherence in the moderately low-income group (poverty level II) and maintained or improved adherence among higher-income groups. Additionally, the prevalence of PA and ST exhibited an overall worsening during this period, with socioeconomic inequalities in PA persisting without substantial change. These findings suggest that the return to the pre-pandemic economic and behavioural conditions has contributed to the re-emergence of dietary disparities and overall deterioration in health behaviours. Sustained public health efforts are essential to address these inequalities and promote healthier lifestyles among vulnerable populations.

## Abbreviations

CIs: Confidence intervals
PA: Physical activity
RII: Relative index of inequality
SII: Slope index of inequality
ST: Screen time
WHO: World Health Organization

## Supplementary information

**Additional file 1:** **e-Table 1.** Missing number and percentage of each variable each survey year. **e-Table 2** Model fitting comparison between Linear and quadratic trend by each health behaviour.

## Declarations

### Availability of data and materials

The data used in this study are available from the Sasakawa Sports Foundation. Researchers can access the data by submitting a web-based application through the foundation’s official website (https://www.ssf.or.jp/thinktank/sports_life/application/index.html).
