# Supplementary material for: Socioeconomic inequalities in health behaviours pre- and post-COVID-19 among Japanese school-aged adolescents: a nationally representative three-wave repeated cross-sectional survey
Source: Environ Health Prev Med. 2025 Sep 11;30:70. doi: 10.1265/ehpm.25-00052 (PMC12436070; doi:10.1265/ehpm.25-00052)
Supplement: Supplementary file 1 — Additional file 1: e-Table 1. Missing number and percentage of each variable each survey year. e-Table 2 Model fitting comparison between Linear and quadratic trend by each health behaviour. [file ehpm-30-070-s001.docx]

Appendix

| e-Table 1. Missing number and percentage of each variable each survey year. | | | | | | | | |
| --- | --- | --- | --- | --- | --- | --- | --- | --- |
|  | 2019 (N = 1076) | |  | 2021 (N = 1025) | |  | 2023 (N=898) | |
|  | n | % |  | n | % |  | n | % |
| sex | 0 | 0.0 |  | 0 | 0.0 |  | 0 | 0.0 |
| age | 0 | 0.0 |  | 0 | 0.0 |  | 0 | 0.0 |
| Residence area | 0 | 0.0 |  | 0 | 0.0 |  | 0 | 0.0 |
| Household income | 239 | 22.2 |  | 232 | 22.6 |  | 232 | 25.8 |
| Family structure | 0 | 0.0 |  | 0 | 0.0 |  | 0 | 0.0 |
| Sports participants | 3 | 0.3 |  | 6 | 0.6 |  | 17 | 1.9 |
| Self-rated health | 4 | 0.4 |  | 3 | 0.3 |  | 6 | 0.7 |
| Preference of physical activity | 5 | 0.5 |  | 4 | 0.4 |  | 9 | 1.0 |
| MVPA | 5 | 0.5 |  | 3 | 0.3 |  | 0 | 0.0 |
| Screen time | 76 | 7.1 |  | 78 | 7.6 |  | 61 | 6.8 |
| Sleep | 10 | 0.9 |  | 7 | 0.7 |  | 7 | 0.8 |
| Breakfast | 1 | 0.1 |  | 3 | 0.3 |  | 5 | 0.6 |
| Bowel movement | 8 | 0.7 |  | 5 | 0.5 |  | 4 | 0.4 |
| *MVPA* moderate-to-vigorous physical activity | | | | | | | | |

| e-table 2 Model fitting comparison between Linear and quadratic trend by each health behavour | | | | | | | | | | |
| --- | --- | --- | --- | --- | --- | --- | --- | --- | --- | --- |
|  |  | Crude SII | | Adjusted SII | |  | Crude RII | | Adjusted RII | |
|  |  | Linear | Quadratic | Linear | Quadratic |  | Linear | Quadratic | Linear | Quadratic |
| Physical activity |  |  |  |  |  |  |  |  |  |  |
|  | AIC | 67.9 | 60.0 | 4133.4 | 4136.0 |  | 66.7 | 60.9 | 1526.4 | 1519.5 |
|  | BIC | 90.5 | 93.9 | 4218.1 | 4232.0 |  | 89.3 | 94.8 | 1611.1 | 1615.5 |
|  | logLik | -29.9 | -24.0 | -2051.7 | -2051.0 |  | -29.4 | -24.4 | -748.2 | -742.7 |
| Screen time |  |  |  |  |  |  |  |  |  |  |
|  | AIC | 65.9 | 64.7 | 4282.1 | 4285.4 |  | 65.7 | 64.6 | 2227.7 | 2228.6 |
|  | BIC | 88.5 | 98.6 | 4366.8 | 4381.4 |  | 88.3 | 98.4 | 2312.4 | 2324.6 |
|  | logLik | -29.0 | -26.3 | -2126.0 | -2125.7 |  | -28.9 | -26.3 | -1098.8 | -1097.3 |
| Sleep |  |  |  |  |  |  |  |  |  |  |
|  | AIC | 61.9 | 64.5 | 4300.9 | 4304.8 |  | 61.9 | 64.5 | 2332.1 | 2335.8 |
|  | BIC | 84.5 | 98.4 | 4385.6 | 4400.8 |  | 84.5 | 98.4 | 2416.8 | 2431.8 |
|  | logLik | -27.0 | -26.3 | -2135.5 | -2135.4 |  | -27.0 | -26.3 | -1151.1 | -1150.9 |
| Breakfast |  |  |  |  |  |  |  |  |  |  |
|  | AIC | 74.4 | 67.3 | 4142.5 | 4145.2 |  | 73.7 | 67.2 | 1677.3 | 1672.2 |
|  | BIC | 97.0 | 101.2 | 4227.2 | 4241.2 |  | 96.3 | 101.1 | 1762.0 | 1768.2 |
|  | logLik | -33.2 | -27.7 | -2056.2 | -2055.6 |  | -32.9 | -27.6 | -823.6 | -819.1 |
| Constipation |  |  |  |  |  |  |  |  |  |  |
|  | AIC | 56.1 | 57.4 | 4063.5 | 4067.4 |  | 56.1 | 59.3 | 1264.4 | 1267.9 |
|  | BIC | 78.7 | 91.3 | 4148.2 | 4163.4 |  | 78.7 | 93.2 | 1349.1 | 1363.9 |
|  | logLik | -24.1 | -22.7 | -2016.7 | -2016.7 |  | -24.1 | -23.6 | -617.2 | -616.9 |
| AIC: Akaike Information Criterion, BIC: Bayesian Information Criterion, logLik: Log-likelihood | | | | | | | | | | |
